# Two Types of Inulin Fructotransferases

**DOI:** 10.3390/ma4091543

**Published:** 2011-09-07

**Authors:** Kazutomo Haraguchi

**Affiliations:** National Food Research Institute, 2-1-12 Kannondai, Tsukuba-shi, Ibaraki 305-8642, Japan; E-Mail: haraguti@affrc.go.jp; Tel.: +81-029-838-8073; Fax: +81-029-838-7996

**Keywords:** inulin, oligosaccharide, DFA III, DFA I, enzyme

## Abstract

Inulin is a polysaccharide contained in chicory, dahlia, and other plants. An oligosaccharide DFA III is produced from inulin using a microbial enzyme, inulin fructotransferase (DFA III producing) [EC 2.4.1.93]. The oligosaccharide DFAIII has a unique functionality that accelerates the assimilation of minerals (Ca, Fe, and so on) from intestines. Therefore, it has a potential for the improvement of osteoporosis and iron deficiency anemia. The production of DFA III was industrialized in 2004 in Japan. Another oligosaccharide DFA I is produced from inulin by another enzyme, inulin fructotransferase (DFA I producing) [EC 2.4.1.200]. The oligosaccharide DFA I has half the sweetness of sucrose. The genes of the two enzymes were cloned and the nucleotide sequences were determined. The deduced amino acid sequences of two enzyme genes had a homology of 49.8%.

## 1. Introduction

In Japan, 600 thousand tons of sucrose is produced annually, from sugar beet produced in Hokkaido. Therefore, the beet sugar production is an important industry of Hokkaido region, even though the consumption of sucrose in Japan is gradually decreasing. Therefore, the introduction of an alternative crop of the sugar beet is expected. In European countries (Germany, Belgium, and so on), a crop chicory has been introduced as an alternative to the sugar beet. The chicory root contains a polysaccharide inulin. The inulin is a polysaccharide contained in chicory, dahlia, Jerusalem artichoke and other plants. The chemical structure of inulin is a β-2, 1 linked fructose polymer terminated with a sucrose residue. In European countries, inulin is used in various foods as a low calorie dietary fiber, for example a component of chocolate.

**Figure 1:**
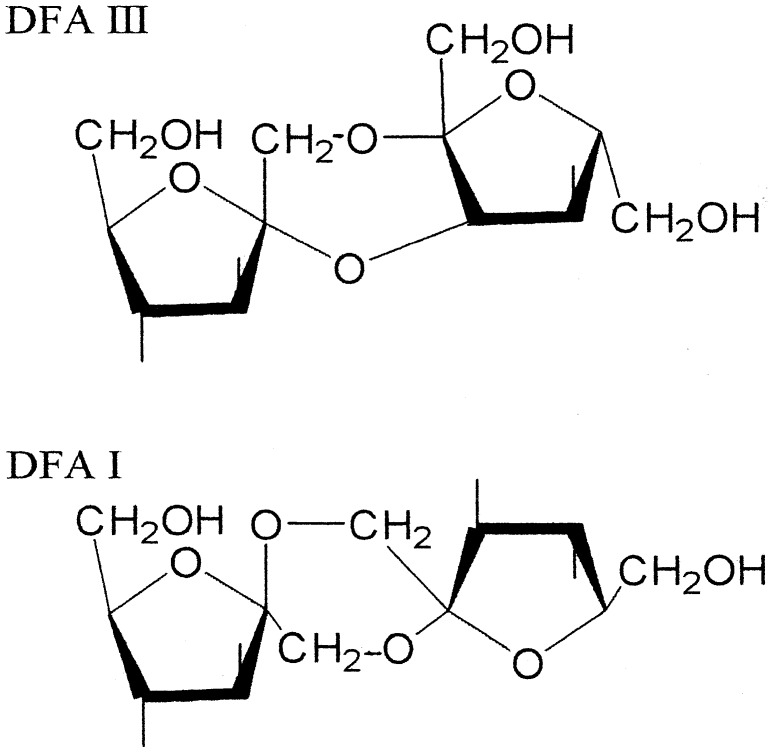
The chemical structures of oligosaccharides DFA III and DFA I.

Using the microbial enzymes, various oligosaccharides are produced from inulin. These oligosaccharides have potential of an application, for example, as a food, a pharmaceutical product, and so on. There are two unique oligosaccharides, DFA III and DFA I, produced from inulin using a microbial enzyme. In DFA III and DFA I, two molecules of fructose are linked to each other at two portions. In this article, we describe two types of enzymes, inulin fructotransferase (DFA III producing), and inulin fructotransferase (DFA I producing). Figure 1 shows the chemical structures of the oligosaccharides DFA III and DFA I.

## 2. Inulin Fructotransferase (DFA III producing)

In studies of inulin decomposing enzymes, inulinases [EC 3.2.1.7] from molds and yeast have been reported in the past. Afterwards, Uchiyama *et al.* (1973) discovered a new type of inulin decomposing enzyme produced by *Arthrobacter* *ureafaciens* [1]. The enzyme converted inulin into an oligosaccharide DFA III (di-D-fructofuranose 1,2’:2, 3’ dianhydride) and a small amount of other oligosaccharides. This DFA III producing enzyme was designated as inulin fructotransferase (DFA III producing) [EC 2.4.1.93]. The enzyme was produced in a culture supernatant of the *A. uereafaciens*. The enzyme was purified with an ammonium sulfate precipitation, an acetone precipitation, and a Sephadex G-100 chromatography. The purity of the enzyme was ascertained by a SDS-PAGE. The enzyme showed maximum activity at pH 6.0 and 50 °C and it was stable up to 50 °C. The molecular mass of this enzyme was estimated to be 80 k Da by a gel filtration.

Subsequently, there have been several reports on the inulin fructotransferase (DFA III producing) from *Arthrobacter* species [2,3,4,5]. Kang *et al.* reported on the enzyme from *Bacillus* sp. [6]. We reported on the enzyme from *Leifsonia* sp. [7]. In many of the DFA III producing enzymes [2,3,4,5,7], the residual oligosaccharides (minor products) are the GF_3_ (nystose) and GF_4_ (fructosyl nystose). Therefore, for these enzymes, the smallest substrate for the enzymes is estimated to be GF_5_.

Sakurai *et al.* (1997) reported on the cloning of the gene of inulin fructotransferase (DFA III producing) from *Arthrobacter* sp. H65-7, for the first time [8]. The genomic library of the *Arthrobacter* H65-7 DNA was screened by colony hybridization and a positive clone was obtained. The cloned 2.2 k bp *EcoR* I-*Kpn* I fragment contained the gene of the enzyme of *Arthrobacter* sp. H65-7. The gene had an open reading frame of 1314 base pairs, and that encoded a signal peptide of 32 amino acids. Therefore, it was estimated that the mature enzyme protein is composed of 405 amino acids.

We reported on the cloning of the gene of inulin fructotransferase (DFA III producing) from *Arthrobacter globiformis* C11-1 [9]. The gene contained a 1353 base pairs open reading frame, and it encoded a signal peptide of 40 amino acids and the mature enzyme protein of 410 amino acids. The deduced amino acid sequence of the enzyme of *A. globiformis* C11-1 had a homology of 74% with that of *Arthrobacter* sp. H65-7.

The DFA III is a non-reducing sugar; mp 164 °C. The DFA III has half the sweetness of sucrose. It was found (2000) that the DFA III accelerates the assimilation of minerals (Ca, Fe, and so on) from intestines [10]. Therefore, the DFA III has a potential for the improvement of osteoporosis and iron deficiency anemia. The production of DFA III was industrialized, using the inulin fructotransferase (DFA III producing) from *Arthrobacter* sp. H65-7 [4]. The sales of the DFA III containing products on the market started in 2004 in Japan. Now DFA III containing commodities are on sale in drugstores and convenience stores in Japan.

## 3. Inulin Fructotransferase (DFA I producing)

We (1989) reported on another type of inulin decomposing enzyme produced by *Arthrobacter globiformis* S14-3, for the first time [11]. The enzyme converted inulin into an oligosaccharide DFA I (di-D-fructofuranose 1,2’:2,1’ dianhydride) and a small amount of the other oligosaccharides. This enzyme was designated as inulin fructotransferase (DFA I producing) [EC 2.4.1.200]. This enzyme is produced in a culture supernatant of *A. globiformis* S14-3. The enzyme was purified by DEAE-Toyopearl chromatography performed in triplicate. The purity was ascertained by a SDS-PAGE. The purified enzyme showed maximum activity at pH 6.0 and 40 °C, and it was stable up to 70 °C at pH 6.0. The molecular mass of the enzyme was estimated as 39 kDa by a SDS-PAGE and 46 kDa by a gel filtration. Therefore, the enzyme of *A. globiformis* S14-3 was considered to be a monomer.

Afterwards, there have been a few reports on inulin fructotransferase (DFA I producing) from *Arthrobacter* species [12,13]. Kushibe *et al.* reported on the enzyme from *Streptomyces* sp. [14].

**Figure 2:**
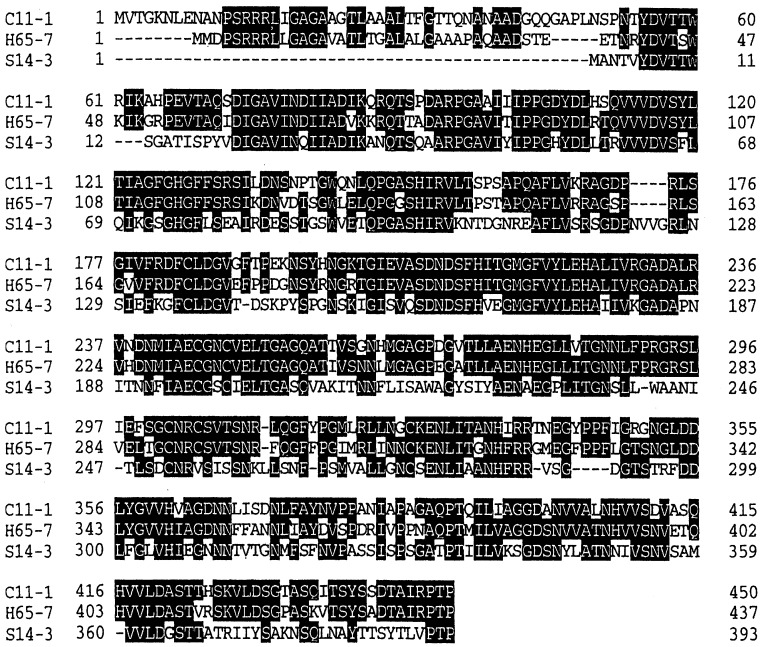
The comparison of deduced amino acid sequences of inulin fructotransferases.

C11-1: Deduced sequence of inulin fructotranferase (DFA III producing) from *Arthrobacter* *globiformis* C11-1; H65-7: deduced sequence of inulin fructotransferase (DFA III producing) from *Arthrobacter* sp. H65-7; S14-3: deduced sequence of inulin fructotransferase (DFA I-producing) from *Arthrobacter globiformis* S14-3.

We (1995) reported on the cloning of inulin fructotransferase (DFA I producing) gene from *Arthrobacter globiformis* S14-3, for the first time [15]. The genomic library of the *A.* *globiformis* S14-3 was screened by colony hybridization and a positive clone was obtained. The cloned 1.5 k bp *Sph* I fragment contained the gene of the enzyme from *A. globiformis* S14-3. The cloned gene had an 1182 bp open reading frame, and it encoded 392 amino acid residues. As mentioned previously, this enzyme is an extra-cellular enzyme produced in the culture supernatant of the microorganism. Though, this enzyme gene has not a structure for a signal peptide. The deduced amino acid sequence of the enzyme had a homology of 49.8% with that of the inulin fructotransferase (DFA III producing) from *Arthrobacter* sp. H65-7 [8]. This result suggests that the inulin fructotransferase (DFA III producing) and inulin fructotransferase (DFA I) have the same genetic origin. Figure 2 shows the comparison of deduced amino acid sequences of the enzymes [8,9,15].

The oligosaccharide DFA I is a non-reducing sugar; mp. 163 °C. It has half the sweetness of sucrose, therefore it has a potential for a new type of a low calorie sweetener.
